# Predisposing factors for allogeneic blood transfusion in patients with rheumatoid arthritis undergoing primary unilateral total knee arthroplasty

**DOI:** 10.3389/fsurg.2023.1205896

**Published:** 2023-07-25

**Authors:** Xiaolin Wang, Liang Zhang, Hongchao Li, Tao Bian, Yixin Zhou, Yujun Li

**Affiliations:** ^1^ Department of Anesthesiology, Beijing Jishuitan Hospital, Capital Medical University, the Fourth Clinical College of Peking University, Beijing, China; ^2^ Department of Orthopedic Surgery, Beijing Jishuitan Hospital, Capital Medical University, the Fourth Clinical College of Peking University, Beijing, China; ^3^ Department of Rheumatology and Immunology, Beijing Jishuitan Hospital, Capital Medical University, the Fourth Clinical College of Peking University, Beijing, China

**Keywords:** rheumatoid arthritis, total knee arthroplasty, allogeneic blood transfusion, risk factors, tranexamic acid

## Abstract

**Background:**

To determine the incidence and identify the predisposing factors for allogeneic blood transfusion (ABT) in patients with rheumatoid arthritis (RA) undergoing primary unilateral total knee arthroplasty (TKA).

**Methods:**

A total of 702 patients with RA who underwent primary unilateral TKA between 2003 and 2022 at a single center, were retrospectively enrolled. Patients were stratified into the ABT and non-ABT groups. Data on patient demographics, laboratory parameters, and disease- and surgery-related parameters were collected from chart reviews and compared between the ABT and non-ABT groups. Multivariate logistic regression analysis was conducted to identify the possible factors associated with postoperative ABT.

**Results:**

A total of 173 (24.6%) patients underwent ABT after surgery. Significant risk factors for ABT included the degree of flexion contracture [odds ratio (OR) = 1.018, *P* = 0.005] and thickness of insertion (OR = 1.170, *P* = 0.014). Conversely, body mass index (OR = 0.937, *P* = 0.018), preoperative hemoglobin level (OR = 0.973, *P* < 0.001), and intraoperative use of tranexamic acid (TXA) (OR = 0.119, *P* < 0.001) were associated with a lower risk of ABT in TKA.

**Conclusion:**

We identified the significant risk and protective factors for ABT during TKA in patients with RA. This information could be helpful in optimizing perioperative blood management strategies during these surgeries.

## 1. Introduction

The acceptance of treat-to-target (T2T) strategies has triggered significant breakthroughs in the treatment of rheumatoid arthritis (RA) (1, 2). A considerable number of patients with RA still suffer from progressive joint damage, which reduces their quality of life and results in the need for arthroplasty in up to 25% of cases (3, 4). Total knee arthroplasty (TKA) provides an opportunity to return to an acceptable quality of life for these patients, while extensive joint damage can lead to excessive blood loss and increase the probability of perioperative blood transfusion (5–7). Compared to the patients with osteoarthritis, patients with RA often have insufficient autologous blood reserves, and therefore, more frequently require allogeneic blood transfusion (ABT) (8–10). Many factors associated with ABT in patients with RA receiving total joint arthroplasty have been reported, including sex, baseline hemoglobin level, arthroplasty type, body mass index (BMI), anesthesia method, and use of tranexamic acid (TXA) (8–10).

Conversely, a series of risks associated with ABT should be considered, including acute transfusion reactions, hemolytic reactions, transfusion-associated circulatory overload and acute lung injury, transmission of blood-borne viruses, iron overload, and graft-versus-host disease. Moreover, patients who undergo ABT tend to have more perioperative adverse events, including mortality and surgical site infections (11, 12). Consequently, it is crucial to understand the risk factors for ABT after TKA in patients with RA.

To the best of our knowledge, no study has yet focused on ABT in patients with RA undergoing TKA. Herein, we conducted a retrospective analysis to determine the incidence of ABT and identify possible associated factors in patients with RA undergoing primary unilateral TKAs at a single high-volume tertiary care center for musculoskeletal diseases.

## 2. Methods

A total of 968 patients with RA who underwent primary unilateral TKA between 2003 and 2022 were retrospectively included in the current study. All patients met the 1987 American College of Rheumatology (ACR) classification criteria or the 2010 ACR/European League Against Rheumatism (EULAR) classification criteria (13, 14). If the patients had undergone staged bilateral TKA, we included only those for whom the operation interval was at least 3 months. The exclusion criteria included simultaneous bilateral TKA, revision surgery, a prior history of infection, trauma, surgery in the operated knee, and autologous blood predonation. This study was approved by the institutional review board of our hospital (No. 202106-57).

Patient demographics included sex, age at TKA, and BMI. The disease-related parameters included the American Society of Anesthesiologists score, rheumatic disease comorbidity index (RDCI) (15), degree of knee flexion, and degree of flexion contracture. Comorbidities were assessed using the RDCI (range 0–9), representing the weighted sum score of seven common comorbidities (15). The surgery-related parameters included the side of operation, type of anesthesia, implant design (low or high constraint), thickness of insertion, intraoperative use of TXA, and postoperative anticoagulation.

Preoperative laboratory parameters included the erythrocyte sedimentation rate (ESR), C-reactive protein (CRP), rheumatoid factor, white blood cell count, hemoglobin (Hb), hematocrit (Hct), platelet (PLT) count, albumin (ALB), prothrombin time (PT), activated partial thromboplastin time, international normalized ratio, and fibrinogen concentration. All data were acquired within 1 week prior to TKA.

All patients underwent TKAs using the standard medial parapatellar approach. Antibiotic prophylaxis was administered intravenously within 15 min prior to tourniquet deflation. Palacos® bone cement was used to fix the implants in all the knees. For patients who were routinely taking aspirin or clopidogrel before surgery, the procedure was performed 1 week after discontinuation. Antibiotics to prevent infection were administrated 24–72 h postoperatively.

Based on our institutional policy, the ABT triggers were set as follows: a Hb level < 7.0 g/dl and Hct < 25%; a Hb level < 9.0 g/dl for patients over 70 years old with severe systemic comorbidities or symptoms of acute anemia (including an altered mental status, low blood pressure, pallor, and shortness of breath not due to other causes).

### 2.1. Statistical analysis

All statistical analyses were performed using SPSS software (version 23.0; Armonk, New York, USA). The normality of continuous data was evaluated using the Shapiro–Wilk test. Data on patient demographics, laboratory parameters, and disease- and surgery-related parameters were collected from chart reviews and compared between the ABT and non-ABT groups. Continuous variables are expressed as the median [interquartile range (IQR)] and were compared using the Mann–Whitney *U* test. Categorical variables were expressed as frequencies and percentages and were compared using Pearson's chi-square test or Fisher's exact test. Multivariate logistic regression was used to investigate factors associated with ABT. The final models for the multivariate analysis were chosen using the forward likelihood ratio method. The odds ratio (OR) with 95% confidence interval (CI) and the associated *P* value were determined. Statistical significance was set at *P* < 0.05.

## 3. Results

A total of 702 patients were enrolled in our study and 173 (24.6%) of these required postoperative ABT. The results of the intergroup comparisons of patient demographics, disease- and surgery-related parameters between the ABT and non-ABT groups are summarized in Table 1. Patients with RA in the ABT group showed lower BMI (22.7 kg/m^2^ vs. 24.0 kg/m^2^, *P* < 0.001), higher degree of flexion contracture (20.0° vs. 15.0°, *P* = 0.008), higher percentage of ASA3 (20.8% vs. 10.4%, *P* = 0.002), higher percentage of general anesthesia (13.9% vs. 5.7%, *P* < 0.001), higher thickness of insertion (10 vs. 10 mm, *P* = 0.003), and a lower frequency of TXA use (23.7% vs. 73.0%, *P* < 0.001).

**Table 1:** Comparison of patient demographics, disease- and surgery-related parameters between the ABT and non-ABT groups.

Variable	ABT group (*n* = 173)	Non-ABT group (*n* = 529)	*P* value
Female, *n* (%)	152 (87.9%)	458 (86.6%)	0.664
Age at TKA, years, median (IQR)	59.0 (50.0, 65.0)	61.0 (53.0, 67.0)	0.058
BMI, kg/m^2^, median (IQR)	22.7 (20.0, 25.4)	24.0 (21.3, 27.1)	**<0.001**
Right side, *n* (%)	91 (52.6%)	271 (51.2%)	0.754
RDCI, median (IQR)	1.0 (0.0, 2.0)	1.0 (0.0, 2.0)	0.832
Degree of flexion (°), median (IQR)	90.0 (85.0, 110.0)	100.0 (90.0, 110.0)	0.148
Degree of flexion contracture (°), median (IQR)	20.0 (10.0, 30.0)	15.0 (0, 28.8)	**0.008**
ASA score, *n* (%)			
1	6 (3.5%)	26 (4.9%)	**0.002**
2	131 (75.7%)	448 (84.7%)	
3	36 (20.8%)	55 (10.4%)	
General anesthesia, *n* (%)	24 (13.9%)	30 (5.7%)	**<0.001**
Implant design, *n* (%)			
Low constraint	164 (94.8%)	511 (96.6%)	0.285
High constraint	9 (5.2%)	18 (3.4%)	
Thickness of insertion (mm), median (IQR)	10.0 (10.0, 12.0)	10.0 (9.0, 11.0)	**0.003**
Postoperative anticoagulation, *n* (%)	155 (89.6%)	487 (92.1%)	0.314
Intraoperative TXA use, *n* (%)	41 (23.7%)	386 (73.0%)	**<0.001**

ABT, allogeneic blood transfusion; TKA, total knee arthroplasty; IQR, interquartile range; BMI, body mass index; RDCI, rheumatic disease comorbidity index; ASA, American Society of Anesthesiologists; TXA, tranexamic acid.

Bold values represent statistical significance.

The results of the intergroup comparisons of laboratory parameters between the ABT and non-ABT groups are summarized in Table 2. The preoperative levels of Hb (115.5 vs. 122.5 g/L, *P* < 0.001), Hct (35.2% vs. 37.4%, *P* < 0.001), and ALB (38.9 vs. 40.6 g/L, *P* < 0.001) were significantly lower in the ABT group than in the non-ABT group. Regarding the RA disease activity and severity of joint damage, patients with RA in the ABT group exhibited significantly higher ESR (50.0 vs. 37.0 mm/h, *P* < 0.001) and CRP (17.7 vs. 13.1 mg/L, *P* = 0.013), while the preoperative PT level (11.6 vs. 11.9 s, *P* < 0.001) was significantly lower in the ABT group than the non-ABT group.

**Table 2:** Comparison of laboratory parameters between the ABT and non-ABT groups.

Variable, median (IQR)	ABT group (*n* = 173)	Non-ABT group (*n* = 529)	*P* value
WBC, ×10^9^/L, median (IQR)	6.40 (5.30, 7.44)	6.57 (5.43, 8.06)	0.101
Hb, g/L, median (IQR)	115.5 (103.0, 122.8)	122.5 (112.0, 132.0)	**<0.001**
Hct, %, median (IQR)	35.2 (32.8, 37.6)	37.4 (34.7, 40.2)	**<0.001**
PLT, ×10^9^/L, median (IQR)	284.0 (230.0, 353.3)	271.0 (215.0, 339.8)	0.170
ESR, mm/1 h, median (IQR)	50.0 (27.0, 79.0)	37.0 (19.0, 59.0)	**<0.001**
CRP, mg/L, median (IQR)	17.7 (6.9, 42.1)	13.1 (4.8, 28.9)	**0.013**
ALB, g/L, median (IQR)	38.9 (36.8, 41.8)	40.6 (37.9, 42.9)	**<0.001**
RF, U/L, median (IQR)	100.5 (24.9, 257.5)	89.4 (23.0, 244.8)	0.347
PT, s, median (IQR)	11.6 (10.8, 12.4)	11.9 (11.3, 12.7)	**0.001**
APTT, s, median (IQR)	27.2 (24.6, 30.3)	27.0 (24.4, 29.8)	0.509
INR, median (IQR)	1.0 (1.0, 1.1)	1.0 (1.0, 1.0)	**0.014**
Fibrinogen, mg/dl, median (IQR)	407.1 (325.7, 477.2)	394.6 (320.2, 468.3)	0.391

ABT, allogeneic blood transfusion; IQR, interquartile range; Hb, hemoglobin; Hct, hematocrit; PLT, platelet; ESR, erythrocyte sedimentation rate; CRP, C-reactive protein; ALB, albumin; RF, rheumatoid factor; PT, prothrombin time; APTT, activated partial thromboplastin time; INR, international normalized ratio.

Bold values represent statistical significance.

Multivariate logistic regression further revealed factors of significance for postoperative ABT which included BMI (OR = 0.937, 95% CI 0.888–0.989, *P* = 0.018), Hb (OR = 0.973, 95% CI 0.960–0.985, *P* < 0.001), degree of flexion contracture (OR = 1.018, 95% CI 1.005–1.032, *P* = 0.005), thickness of insertion (OR = 1.170, 95% CI 1.032–1.326, *P* = 0.014), and intraoperative use of TXA (OR = 0.119, 95% CI 0.079–0.180, *P* < 0.001, Table 3).

**Table 3:** Results of multivariate logistic regression analysis of ABT in TKA.

Variable	OR	95% CI	*P* value
BMI	0.937	0.888–0.989	0.018
Degree of flexion contracture	1.018	1.005–1.032	0.005
Hb	0.973	0.960–0.985	<0.001
Thickness of insertion	1.170	1.032–1.326	0.014
Intraoperative TXA use	0.119	0.079–0.180	<0.001

ABT, allogeneic blood transfusion; TKA, total knee arthroplasty; OR, odds ratio; CI, confidence interval; BMI, body mass index; Hb, hemoglobin; TXA, tranexamic acid.

## 4. Discussion

To the best of our knowledge, this is the first study to retrospectively assess the incidence of postoperative ABT in patients with RA undergoing TKAs. We reported an incidence of 24.6% of postoperative ABT in these patients. Body mass index, degree of flexion contracture, preoperative Hb level, insertion thickness, and intraoperative use of TXA were all found to be associated with postoperative ABT.

Previous studies have reported a relatively high incidence of ABT in patients with RA undergoing THAs and TKAs (8–10). Morse et al. (8) retrospectively reviewed the cases of 252 patients with RA. In this cohort, 146 participants (57.9%) underwent TKAs, while 106 (42.1%) underwent THAs. The overall transfusion rate after surgery was 11.2%. In another retrospective study by Ogbemudia et al. (9), 349 patients with RA were reviewed. These patients underwent either THAs of TKAs at a single university teaching hospital, and 21% (*n* = 72) required ABT. A significant preoperative factor associated with postoperative ABT was the THA procedure (*P* = 0.008). Salt et al. (10) conducted a retrospective study (*n* = 3,270) in patients with RA following total joint arthroplasty (knee, 65.5%; hip, 30.2%; and shoulder: 4.3%). The overall transfusion rate after surgery was 19.9%. They also found that patients who received THAs were more likely to experience ABT compared to TKAs (OR = 1.39; 95% CI, 1.14–1.79; *P* = 0.001). In contrast, the incidence of postoperative ABT in our study was relatively higher (24.6%). We believe that this trend could be explained by several factors. First, in this study, we exclusively enrolled patients who received TKAs, and theoretically, the incidence of postoperative ABT should be lower than that in the results mentioned above, considering that the intraoperative use of tourniquets may significantly control the amount of visible intraoperative blood loss. Furthermore, a complete removal of the hypertrophied synovial villi and pannus is necessary for the relief of symptoms and control of postoperative flare. Therefore, hidden blood loss after TKA with a tourniquet is a concern in this special patient population. Second, the time span of the current study was relatively large (2003–2022). In the early stage of this time period, the intraoperative use of TXA was not popularized in clinical practice, which had a strong impact on the actual incidence of postoperative ABT.

Most of the relevant studies have supported the notion that anemia was a key risk factor for ABT in patients with RA who underwent total joint arthroplasty, which is consistent with the findings of previous studies. One study found anemia to be one of the most common extra-articular manifestations of RA (16). Wilson et al. (17) reviewed English-language articles on anemia in RA and published in the pre-biotherapy era (1996–2001) between 1966 and 2001. This review found that 4.5% of patients had Hb levels lower than 8 g/dl during joint replacement surgery. Moderate anemia (defined in a number of ways) was found in 33.3%–59.1% of patients; the upper limit of normal was set at 14 or 12 g/dl, and the lower limit of normal at 9.5 or 10 g/dl. In a prospectively acquired database of 2,120 patients with RA (18), the prevalence of anemia, as defined by the WHO, was 30.4% in men and 32.0% in women. This prevalence was three times higher in patients with RA than in 7,124 patients without inflammatory joint disease. Hb levels were lower than 10 g/dl in 3.4% of patients with RA and lower than 11 g/dl in 11.1% of these patients. The causes of anemia in RA include chronic inflammation, iron deficiency anemia, folate deficiency anemia (usually in patients taking methotrexate), vitamin B12 deficiency anemia, hemolytic anemia, and anemia related to myelodysplastic syndrome (16). The key target of perioperative blood management in patients with RA is to eliminate the need for ABT, while simultaneously preventing anemia. The best means of correcting anemia in chronic inflammation is to ensure systemic disease control by administering conventional synthetic disease-modifying antirheumatic drugs (csDMARDs), particularly methotrexate (MTX), biological disease-modifying antirheumatic drugs (bDMARDs), and targeted synthetic disease-modifying antirheumatic drugs (tsDMARDs). The use of iron supplementation, erythropoietin, and preoperative autologous blood donation may also be considered as supplementation if necessary.

Body mass index is regarded as a risk factor for transfusion in the general patient population (19–21), but not in RA patients undergoing arthroplasty (8–10). Clinically, a low BMI tends to indicate combined osteoporosis in patients with RA, which may contribute to an increased perioperative hidden blood loss. In addition, low BMI is more commonly observed in patients with juvenile-onset RA. These patients are prone to severe bone deformities and defects, compromised bone quality, malalignment, and contracture as a result of premature growth plate closure during childhood. Because of the altered anatomy of the bony and soft tissue structures, the knees in such patients may be reconstructed by highly constrained or even customized implants, which could inevitably add to the need for ABT. Regarding the other two significant factors, the degree of flexion contracture and thickness of insertion were also closely associated with the degree of local deformities of bony and soft tissue structures and the complexity of surgical construction. The existence of these disease- and surgery-related factors in association with increased technical challenges may prompt surgeons to plan more active solutions for excessive intraoperative blood loss, including preoperative autologous blood donation and intraoperative cell saver usage.

In recent years, the intraoperative use of TXA in THA/TKA has been well accepted, and its efficacy and safety in decreasing intraoperative blood loss has been confirmed (22–24). Only one RA-specific study by Morse et al. (8) failed to observe a lower transfusion rate in patients who received TXA, and the authors suggested that this result may be due to the underlying disease process itself, or the baseline anemia in these special cases. The results of the current study revealed that intraoperative use of TXA was significantly less frequent in the ABT group than that in the non-ABT group (23.7% vs. 73.0%), and patients who were administered TXA had an 8.4-fold decreased need for postoperative ABT (OR = 0.119, 95% CI 0.079–0.180, *P* < 0.001). Consequently, we recommend the intraoperative use of TXA during TKA in patients with RA, although further research on its safety should be conducted.

This study had some limitations. First, due to the retrospective observational nature of this study, there was inevitably some missing valuable information, although the sample size was relatively large and all cases were from a single, high-volume tertiary care center for musculoskeletal diseases. Some confounders, such as baseline disease activity score of 28 joints (DAS28) and simple disease activity index as indicators of disease activity, preoperative medications, operation time, accurate intraoperative bleeding, the use of cell salvage, and postoperative drainage, were not adequately adjusted. Second, the time span of the study was almost two decades (2003–2022), and we failed to trace the dynamic trend of postoperative ABT. During this period surgical theory and prosthesis design had greatly changed, which inevitably led a bias within the results. Third, there was a lack of a control group; therefore, no conclusions can be drawn regarding the differences between patients with RA and osteoarthritis.

## 5. Conclusion

In conclusion, we reported a relatively high incidence of postoperative ABT in patients with RA and identified significant patient demographics, laboratory parameters, and disease- and surgery-related parameters for postoperative ABT. Taking these modifiable factors into consideration is an important step toward establishing an effective perioperative blood management strategy to minimize the need for ABT and decrease the rate of perioperative adverse events in clinical practice.

## Data Availability

The raw data supporting the conclusions of this article will be made available by the authors, without undue reservation.
